# Post-Traumatic Stress Disorder, Major Depressive Disorder, and Wildfires: A Fifth-Year Postdisaster Evaluation among Residents of Fort McMurray

**DOI:** 10.3390/ijerph19159759

**Published:** 2022-08-08

**Authors:** Wanying Mao, Medard Adu, Ejemai Eboreime, Reham Shalaby, Nnamdi Nkire, Belinda Agyapong, Hannah Pazderka, Gloria Obuobi-Donkor, Ernest Owusu, Folajinmi Oluwasina, Yanbo Zhang, Vincent I. O. Agyapong

**Affiliations:** 1 Department of Psychiatry, University of Alberta, Edmonton, AB T6G 2B7, Canada; 2 Global Psychological E-Health Foundation, Edmonton, AB T6G 2B7, Canada; 3 Department of Psychiatry, Faculty of Medicine, Dalhousie University, 5909 Veterans Memorial Lane, 8th Floor, Abbie J. Lane Memorial Building, QEII Health Sciences Centre, Halifax, NS B3H 2E2, Canada

**Keywords:** MDD, PTSD, wildfire, natural disaster, trauma, mental health, Fort McMurray

## Abstract

**Background:** Over 90,000 residents had to be evacuated from Fort McMurray (FMM), Alberta, Canada due to the wildfire that engulfed the city in May 2016. Overall, about 2400 homes or 10% of the housing stock in Fort McMurray were destroyed. The fire consumed about 200,000 hectors of forest, reaching into Saskatchewan. During major disasters, communities’ infrastructure is disrupted, and psychological, economic, and environmental effects are felt for years afterwards. **Objective:** Five years after the wildfire disaster, this study assessed the prevalence rate of major depressive disorder (MDD) and post-traumatic stress disorder (PTSD) in Fort McMurray residents and determined the demographic, clinical, and other risk factors of probable MDD and PTSD. **Methodology:** A quantitative cross-sectional survey was conducted to collect data through an online questionnaire administered via REDCap between 24 April and 2 June 2021. The Patient Health Questionnaire (PHQ-9) was used to assess the presence of MDD symptoms in respondents. The PTSD Checklist for DSM-5 (PCL-C) was used to assess likely PTSD in respondents. Descriptive, univariate, and multivariate regression analyses were employed. **Results:** 186 out of 249 individuals who accessed the survey link completed it (74.7% response rate). The median age of the subscribers was 42. The sample included a majority of 159 (85.5%) females; 98 (52.7%) > 40 years of age; 175 (94.1%) employed; and 132 (71%) in a relationship. The overall prevalence of MDD symptoms in our study sample was 45.0% (76). Four variables independently predicted MDD symptoms in the multivariate logistic regression model, including: unemployed (OR = 12.39; 95% CI: 1.21–126.37), have received a mental diagnosis of MDD (OR = 4.50; 95% CI: 1.57−12.92), taking sedative-hypnotics (OR = 5.27; 95% CI: 1.01−27.39), and willingness to receive mental health counseling (OR = 4.90; 95% CI: 1.95–12.31). The prevalence of likely PTSD among our respondents was 39.6% (65). Three independent variables: received a mental health depression diagnosis from a health professional (OR = 4.49; 95% CI: 1.40–14.44), would like to receive mental health counseling (OR = 4.36, 95% CI: 1.54–12.34), and have only limited or no support from family (OR = 11.01, 95% CI: 1.92–63.20) contributed significantly to the model for predicting likely PTSD among respondents while controlling the other factors in the regression model. **Conclusions:** According to this study, unemployment, taking sleeping pills, having a prior depression diagnosis, and the willingness to receive mental health counseling significantly increase the odds of having MDD and PTSD following wildfires. Family support may protect against the development of these conditions.

## 1. Introduction

Natural disasters impact many people throughout the world, with the changing climates and rising population density on earth increasing the frequency of these disasters [1]. On average, the world experiences major disasters each day, and over 150 million people are deeply affected by disasters annually [1]. Almost half of Canadians report having experienced a significant disorder in their lifetime, 1% have experienced major residential fires, and 3% have been exposed to wildfires [2]. With climate change and development of fire-prone landscapes, wildfires are among the most serious threats to property and life worldwide [3]. Globally, Wildfires and Volcanic Activities have impacted an estimated 6.2 million people since 1998 according to the World Health Organization (WHO) [4]. Statistics currently show that wildfires continue to have significant global impacts. By October 2020, for example, 8 million acres of ground in the United States had burned due to wildfires [5]. Canadians have spent an average of 800 million dollars a year on wildfire-related costs in recent years [6]. Additionally, Australia has experienced an increase of 30.6 days in the number of days with a high–extreme fire danger between 2016 and 2019 [7]. Climate change is causing high temperatures, prolonged summers, and blistering heat waves, all contributing to an increase in wildfire awareness and activity [8]. As a result of the devastating wildfires in Chile, Australia, and California, the global community has been reminded of the destructive effects of uncontrolled fire [3].

Apart from destroying property and causing death and physical injury, wildfires can also result in lasting mental health illnesses, including major depressive disorder (MDD) and post-traumatic stress disorder (PTSD) [1,9,10,11,12,13,14,15,16,17,18,19]. In the years following a natural disaster, major depressive disorder (MDD) is among the most often studied and screened mental illnesses [20]. According to the DSM 5, to diagnose MDD, at least five of the following symptoms have to be present during the same 2-week period (and at least one must be Diminished interest/pleasure or Depressed mood): Depressed mood, Diminished interest, Significant weight change, Sleep disturbance, Psychomotor agitation or retardation, Fatigue or loss of energy, Feelings of worthlessness, Diminished ability to think or concentrate, and Recurrent thoughts of death [21]. Studies evaluating the prevalence of MDD after wildfire show that the percentage of individuals who experience depressive symptoms ranges from 4.9% to 54% [22]. In adults, depression and its associated symptoms have been found to be more common after a fire, which can last up to ten years [23,24,25]. Moreover, adolescents and children have been examined for postwildfire depression rates. In research studies, depression rates after wildfires increase by 20% after six months [22,26]. One third of grade 7–12 students within 18 months of the wildfire met the depression criteria, compared with 17% of students in an age-matched control group [10,27]. Based on the findings of a recent scoping review, it appears that the prevalence of PTSD in communities affected by wildfires is growing statistically and clinically [25]. For PTSD, the criteria requires a certain type and level of traumatic event, a combination of required symptoms, and the absence of exclusionary criteria [28]. Within the first few years after a wildfire and for up to ten years afterwards, adults experienced a higher rate of post-traumatic stress disorder and related symptoms [24,29]. In a cross-sectional study to examine the predictors of higher levels of depression and PTSD symptoms in 1468 adolescents 6 months after a wildfire in Greece, 20.0% of participants reported probable depression, and 29.4% of participants reported having probable PTSD symptoms [30]. Likewise, the study found that residents of the high-affected communities reported more likely incidents of PTSD than residents of the medium and low-affected communities three to four years after the Black Saturday bushfires in the state of Victoria, Australia [31]. Despite that, some studies examining mental health issues in community samples estimated that only about 15% of adults who had suffered at least one life-threatening event developed mental illness [32,33]. Thus, triggers beyond the disaster itself are worth investigating to explain the individual differences

Increased living close to nature and in once-forested areas increases the likelihood of wildfires still raging and posing a threat to humanity [25]. Adequate knowledge and understanding of the risk factors for the development of mental health issues after wildfire disasters can help all departments concerned, such as clinicians, policy makers, public health experts, etc., to provide suitable preventative measures to avoid these symptoms and implement strategies that can rebuild the ability of patients to recover from wildfire.

Following a disaster, people with mental health problems before the disaster report higher incidence rates of mental health issues after a disaster. Among such, major depressive disorder and post-traumatic stress disorder (PTSD) are prominent, but there are also symptoms of panic disorder, generalized anxiety disorder (GAD), psychophysiological stress syndrome (PPSS), and overall increased substance use disorder (SUD) [34]. What is most concerning is the possibility of an increase in sexual violence [35].

The onset of the spring in 2016 throughout northeastern Alberta created conditions for extreme fire behavior in the widespread boreal forest that surrounds Fort McMurray, which caused one of the worst natural disasters in Canadian history [30]. Over 2400 structures and an area of land of approximately 5890 km^2^ were destroyed by the wildfire [36]. Over 90,000 residents of Northern Alberta were relocated as a result, forcing the greatest evacuation in Alberta history—some of whom have never returned [30].

This study was conducted five years after the Fort McMurray wildfire to demonstrate and evaluate the impact of the 2016 Fort McMurray wildfire on the survivors’ mental health. Accordingly, we studied the likelihood of MDD and PTSD among participants using self-administered questionnaires to analyze the possible demographic, clinical, and other risk factors with relationship status as a critical variable among the respondents 5 years after the devastating wildfire. Giving that most of the residence travel from around the country into FMM to work in the oil industry, we were also interested in whether participants varied by relationship status, as anecdotal evidence suggested it is much easier for single individuals to travel from other provinces to work in the oil sand industry. Furthermore, relationship status is a key determinant of mental health.

## 2. Materials and Methods

### 2.1. Study Setting 

Fort McMurray is a city in Northern Alberta, Canada, in the Regional Municipality of Wood Buffalo, with a varied population of 111,687 as of the 2018 census [37]. Due to catastrophic flames that threatened lives and property, Fort McMurray and adjacent communities were ordered to evacuate on 3 May 2016. Approximately 2400 structures in Fort McMurray were destroyed, and over 90,000 people were evacuated from the city and neighboring areas. The evacuation order was rescinded on 1 June 2017, with inhabitants gradually returning to the community [30].

### 2.2. Study Design, Data Collection, and Ethical Consideration

This was a quantitative cross-sectional survey. Data were collected between April 24 and 2 June 2021 through a self-administered online questionnaire via REDCap, a secure browser-based application for building and managing online surveys and translational research databases [38], five years after the Fort McMurray wildfire. This survey was sent randomly via email to the residents in Fort McMurray, Canada, using government, school, occupational, and community platforms to recruit participants. Sociodemographic, clinical, and Fort McMurray wildfire-related questions as well as the level of support received from different jurisdictions were included in the survey. 

In total, 249 residents clicked on the online survey after receiving the invitation from community partners. However, due to none or incomplete survey responses by some of the residents, data from only 186 respondents were included in the final analysis, providing an overall completion rate of 74.7%. However, due to none or partial survey responses from certain individuals, data from just 186 respondents were included in the final analysis, yielding an overall completion rate of 74.7%. The Patient Health Questionnaire (PHQ-9) [39,40] is a self-administered scale designed to measure respondents’ presence of MDD symptoms. It is an effective tool for patients’ population, however, from the literature, it is also perceived as a validated tool for screening of the depressive symptoms among the general population [40]. The PHQ-9 score was calculated according to the standard recommendations, and the depression criterion was fulfilled if 5 of the 9 items were observed at least “greater than half the days” and either item A or B was noticed at least “more than half the days”. The tool’s reliability and validity have shown that it has good psychometric qualities. The PHQ-9 has a high internal consistency: Cronbach alphas of 0.86 and 0.89 were obtained in a study that included two separate patient populations [39]. The PTSD checklist civilian version (PCL-C) [41] is a self-administered scale intended to measure respondents’ likelihood of having PTSD. There were 17 questions in total, with responses ranging from “significantly agree” to “do not agree at all”. People undergoing a PCL-C score of 44 or higher were considered to have PTSD [41]. The PCL-C had an excellent reliability (Cronbach’s alpha = 0.90). According to ROC analysis, a cut-off score of 26 provided optimal discriminating power, with a sensitivity of 0.86 (95% CI: 0.78–0.92) and a specificity of 0.63 (95% CI: 0.62–0.65) [42]. Several investigations found that the PCL-C has strong internal consistency and retest reliability. When compared with other PTSD measures, the PCL-C demonstrated excellent patterns of convergent and discriminant validity. As a result, it is proposed in clinical practice and research as a psychometrically valid tool for screening and measuring the severity of PTSD42 [43,44].

Participants were provided with information about the study, and completing the survey questions implied informed consent. Study approval was granted by the University of Alberta Research and Ethics Committee (Pro00066054).

### 2.3. Sample Size Estimation 

With a population of 111,687 as of the 2018 census, a 95% confidence interval, and a ±3% margin of error, the sample size needed for prevalence rate estimates for MDD and PTSD was 1058.

### 2.4. Statistical Analysis 

Data were analyzed using SPSS Version 25 (IBM Corp 2011, Armonk, NY, USA) [45] with relationship status as a critical variable. Descriptive statistics for demographic, clinical, and wildfire factors versus relationship status were reported. The relationship status was collected initially as three categories including *Married/partnered/cohabiting; Divorced/Separated/Widowed and Single*. Due to the low responses of the last two categories, we opted to combine them in one category. Cross-sectional analyses with chi-squared tests investigated connections, categorical variables, and the probability that respondents had MDD and PTSD. We reported the results without imputed missing data. On univariate analysis, variables with a statistically significant relationship (*p* ≤ 0.05) or approaching significance (0.05 < *p* ≤ 0.1) to the likelihood of MDD and PTSD were included in a logistic regression analysis. Correlational analysis was performed prior to running logistic regression to identify any strong intercorrelations (Spearman’s correlation coefficient of 0.7 to 1.0 or 0.7 to 1.0) among predictor variables. The odds ratios and confidence intervals from the binary logistic regression analysis were examined to determine the relationship between each component in the model and the likelihood of respondents presenting with likely MDD and PTSD while controlling for the other factors.

## 3. Results 

Of the 249 returned survey forms from Fort McMurray’s residents, we included 186 respondents who completed all key psychometric questionnaires in analysis, yielding a completion rate of 74.7%. In total, 76 (45.0%) of the respondents reported moderate to severe MDD symptoms. At the same time, the prevalence of likely PTSD in our study sample was 65 (39.6%).

Descriptive demographic, clinical, and wildfire-related responses were collected from the participants (N = 186), with relationship status as a critical variable (Table 1). The sample included a majority of 159 (85.5%) females; 98 (52.7%) > 40 years of age; 175 (94.1%) employed; and 132 (71%) in a relationship. Regarding the related clinical data, 58 (31.4%) of our respondents had a history of depression diagnosis; 66 (35.5%) reported on medication for mental health concerns; 114 (61.3%) respondents received mental health counseling in the past year; and 98 (52.7%) of the respondents would like to receive mental health counseling. In terms of the wildfire-related and support information, 176 (94.6%) of the respondents resided at FMM during the 2016 wildfire. Respondents reported receiving a some-to-high level of support from family were 151 (86.3%); from the Red Cross were 130 (74.3%); from the Government of Alberta were 109 (62.6%); and from the insurer were 126 (72.4%). More detailed characteristics of respondents are presented in Table 1. 

### 3.1. Univariate Analysis

A summary of the results of the univariate analysis of the relationship between likely MDD and PTSD in respondents and demographic, clinical, and wildfire-related experiences is summarized in Table 2. Fourteen variables were significantly related to moderate-to-high MDD symptoms, and 17 variables were significantly related to likely PTSD. Respondents who were not employed were more likely to present with probable MDD (90%) and PTSD (100%) than those who were employed. Moreover, respondents with a history of depression (MDD: 70.4%; PTSD: 67.3%), an anxiety disorder (MDD: 62.0%; PTSD: 59.45%), and who had history of depressive disorder diagnosed by mental health professional prior to 2016 wildfire (MDD: 58.6%; PTSD: 54.8%) were more likely to present with probable MDD and PTSD symptoms compared with respondents with no history of depression, an anxiety disorder or no mental health diagnosis before the wildfire, respectively. Additionally, respondents using antidepressants (MDD: 59.3%; PTSD: 56.9%), benzodiazepines (MDD: 100%; PTSD: 100%), sleeping tablets (MDD: 84.2%; PTSD: 76.5%), or any medication for mental health concerns before the wildfire (MDD: 59.0%; PTSD: 53.4%) were more likely to develop MDD and PTSD than respondents who were not on antidepressants, benzodiazepines, and sleeping tablets or no psychotropic medication, respectively.

Similarly, participants who underwent mental health counseling treatment (MDD: 57.8%; PTSD: 62.3%) and would wish to obtain mental health counseling (MDD: 62.9%; PTSD: 59.3%) were more likely to present with probable MDD and PTSD symptoms after the wildfire than respondents who did not receive any counseling and would not want to receive mental health counseling. Furthermore, respondents who report fear for their own or their friends’ or family’s lives on the day of the evacuation (MDD: 48.0%; PTSD: 43.4%), and who reported they received limited or no support from a family and Red Cross (Family: MDD: 70.8%; PTSD: 77.3%. RedCross: MDD: 59.5%; PTSD: 53.8%.), were more likely to present with probable MDD and PTSD symptoms than those who reported receiving unconditional support from family and Red Cross, respectively. Furthermore, respondents who reported a loss of property in the fire (MDD: 49.0%; PTSD: 48.0%), homes suffered substantial smoke damage in the wildfire (MDD: 45.5%; PTSD: 57.1%), who lived in a different home after the fire (even if the fire did not destroy the home they lived in before the fire) (MDD: 62.5%; PTSD: 47.8%), and those who reported limited or no support from insurers present with probable PTSD symptoms (52.3%) compared with respondents who did not undergo loss of property in the fire, who lived in the same homes they lived in before the fire, and those reported receiving high-level support from their insurers. Finally, respondents who reported they received no support from the Government of Alberta were more likely to present with MDD symptoms (58.1%) compared with respondents who reported they received a high level of support from the Government of Alberta.

### 3.2. Logistic Regression

Table 3 depicts the multivariable binomial logistic regression model used to determine the likelihood of moderate-to-severe MDD symptoms among the study respondents. We found 14 variables with significant or near-significant *p* values (*p* ≤ 0.1) (Table 2). Eleven of the predictor variables were included in the model, “Never received a mental health diagnosis from a health professional” and “On antidepressant medication” were excluded from the regression model since they were strongly positively correlated with other variables (rs > 0.7). We also removed the variable of “On Benzodiazepines” based on low variability. 

The logistic model was statistically significant; Χ^2^ (n = 167) = (59.54, *p* ≤ 0.001), showing that the model could distinguish respondents who had moderate-to-severe MDD symptoms from those with mild depression during the Fort McMurray wildfire. The model accounted for 30.0% (Cox and Snell R^2^) to 40.1% (Nagelkerke R^2^) of the variance, indicating the likelihood that respondents will present with MDD and accurately identified 76.0% of cases.

As indicated in Table 3, only four variables; unemployed, received a mental diagnosis of MDD, history of receiving sleeping tablet medication (sedative–hypnotics), and willingness to receive mental health counseling independently predicted MDD symptoms in the model. When compared with those who were employed, those who reported being unemployed were more than 12 times more likely to have moderate-to-severe MDD symptoms (OR = 12.39; 95% CI: 1.21–126.37).

When compared with individuals without a history of MDD diagnosis, study participants who had a history of mental health diagnosis were four times more likely to have moderate-to-severe MDD symptoms (OR = 4.50; 95% CI: 1.57–12.92). Respondents who were willing to receive mental health counselling were five times more likely than those who were not willing to receive mental health counseling to have moderate-to-severe MDD symptoms (OR = 4.90; 95% CI: 1.95–12.31). Finally, survey respondents who were using sleeping tablets were five times more likely to have moderate-to-severe MDD symptoms than those who did not use sleeping tablets (OR = 5.27; 95% CI: 1.01–27.39).

The logistic regression model used to predict the likelihood of PTSD symptoms among study participants is shown in Table 4. We found 17 variables with significant or near-significant *p* values (*p* ≤ 0.1) (Table 2). “Never received a mental health diagnosis from a health professional” and “On antidepressant medication” were removed from the regression model as they were highly positively correlated with other variables (rs > 0.7). Additionally, we removed the variables of “Employment” and “On Benzodiazepines” based on low variability.

Therefore, we incorporated 13 variables into the logistic regression model to forecast the likelihood of PTSD. The entire model was statistically significant, X^2^ (162) = (77.32, *p* ≤ 0.001), demonstrating that the model could distinguish between respondents who had likely PTSD and those who did not 5 years after the wildfire. The model accurately classifies 77.2% of the case and correctly classifies 38.0% (Cox and Snell R^2^) and 51.3% (Nagelkerke R^2^) of the variance indicating the likelihood that respondents will present with PTSD. 

Table 4 shows that only three independent variables contributed significantly to the model for predicting likely PTSD among respondents: having a mental diagnosis of MDD, being willing to attend mental health counselling, and having limited or no support from family. Respondents with a history of MDD mental health diagnosis were four times more likely to have probable PTSD than those who did not (OR = 4.49; 95% CI: 1.40–14.44). Similarly, participants who were willing to receive mental health counselling were more than four times as likely than those who were not willing to receive mental health counselling to express PTSD (OR = 4.36; 95% CI: 1.54–12.34). Furthermore, individuals who reported receiving little or no support from family after the wildfire were 11 times more likely to experience likely PTSD symptoms than those who received moderate-to-strong family support (OR = 11.01; 95% CI: 1.92–63.20).

## 4. Discussion

This research used a self-administered survey five years after the Fort McMurray wildfire in 2016 to assess the prevalence and potential predictors of likely MDD and PTSD among Fort McMurray residents who participated in the study. The overall prevalence of MDD in our survey stood at 45.0%, and the prevalence of likely PTSD was 39.6% among FMM residents. As noted above, the prevalence of MDD is higher than the 18.3% prevalence found among school staff of Fort McMurray eighteen months after the May 2016 wildfire and also higher than the estimated lifetime prevalence of MDD (9.7%) and (12.6%) in the population of Alberta and Canada, respectively [46]. As well, the prevalence found in our study is higher than other previous surveys on community-based wildfires such as the three to four years after the Victoria Black Saturday bushfire and the three months post-2003 California firestorm, which reported rates of 12.9% [31] and 33% [23] for MDD, respectively. Similarly, the prevalence of likely PTSD in our study was high when compared with the prevalence of 12.8% and 13.6% in the Fort McMurray general population sixteen months and eighteen months after the wildfire, respectively [30,36]. In this way, the devastation caused by wildfires often persists for an extended period, resulting in further disruption of daily functioning, as well as psychological adjustment problems and diminished well-being [23,47]. 

### 4.1. Major Depression Disorder 

Unemployed respondents were almost 10 times more likely than employed respondents to present with likely MDD. The association between unemployment and poor mental health has long been studied and documented in the literature [48,49,50,51]. Reduced psychosocial wellbeing, as well as life satisfaction in individuals, is deemed to be linked to unemployment, which in turn raises the likelihood of acquiring affective disorders [50]. Our finding is consistent with a prior research that assessed the relationship between unemployment and MDD, with the prevalence of depression among the unemployed population rated at 24% [52]. Another possible explanation of the association between likely MDD and unemployment is that unemployed individuals have a higher rate of indulging in unhealthy behaviors and lifestyles, which in turn may raise the risk of MD [53,54].

Results found in our study show that having a history of depression diagnosis predicted the likelihood of developing MDD symptoms following experiences of wildfire in respondents. That means participants who answered “yes” to having received a mental health diagnosis of depression in the past were four times more likely to have likely MDD than those who did not. Our results are consistent with prior research that aimed to assess the patterns and predictors of depressive symptom trajectories over time following mass traumatic events [1,36,55,56,57,58]. According to our findings, respondents who reported a lifetime history of MDD were more likely to present with MDD postwildfire than respondents who did not report a lifetime history of MDD. Our findings, on the other hand, contradict those of a previous study conducted in a similar setting six months after the Fort McMurray catastrophe, which demonstrated that having a history of depression was not a significant predictor of subsequent MDD symptoms after the disaster [59].

We also observed that among participants who took sleep medication, there was a fivefold increase in moderate to severe depression five years after the wildfire compared with those who did not take sleep medication. This observation is consistent with a recent study that looked at characteristics that influence the diagnosis of depression in older primary care patients [60]. In this study, the researcher found that the presence of MDD symptoms was associated with patients who acknowledged taking sedative–hypnotic medication (OR = 2.6, 95% CI = 1.3–5.4) [60]. A possible explanation is that, since sleep is a key determinant of an individual’s overall health and well-being and a critical health-related factor, sleep plays a major role in the development of many disorders, including MDD. Studies have suggested that chronic sleep deprivation increases the risk of developing MDD [61,62], and people who report routinely obtaining little or no sleep have an unusually high prevalence of MDD [60,63].

In addition, our research findings demonstrate that following the wildfire, the willingness to seek mental health counseling predicted the chance of acquiring likely MDD. It implies that respondents who were willing to receive mental health counseling were four times more likely than those who were not willing to receive mental health counselling to present with MDD symptoms. This outcome is congruent with the findings of a previous study, which looked at the relationship between college students’ mental health and psychological help-seeking behavior during the COVID-19 pandemic [64]. The results of the study imply that respondents who sought psychological assistance had higher MDD prevalence [64]. This suggests that people with mental health conditions such as MDD often resort to counseling as they perceive worse psychological well-being during or after the occurrence of traumatic events. It further suggests that psychologically minded individuals are in-tune with their mental health needs and are perhaps more likely to identify and report mental health distress (including MDD symptoms) and seek psychological therapies [65].

Our study further discovered that some wildfire-related variables which were not predictive of MDD five years after the wildfire were predictive of MDD 18 months after the wildfire. Thus, respondents’ perceptions of having received limited or no support from family had a 52.6% (*p* = 0.00) impact on the likelihood of MDD diagnosis 18 months after the wildfire [46] but was not predictive of MDD five years on. The possible explanation may be that failure to immediately mobilize support from the family goes a long way to affecting the psychological well-being of victims with the potential of developing depression in the short-to-medium term. This notwithstanding, the victims may be able to build resilience in the long term, which may have resulted in the failure of these variables to predict likely MDD five years on.

### 4.2. Post-Traumatic Stress Disorder

The meta-analysis carried out by Ozer and colleagues classified PTSD-related predictors/risk factors into seven groups, which included prior history of trauma, previous psychological problems, family history of mental illness, perceived life threat, post-trauma social support, peritraumatic emotional responses, and peritraumatic dissociation [66]. Part of our findings in this research overlap significantly with these categories—prior depression history and limited or no social support. 

According to our study findings, subscribers who reported having a history of depression before the wildfire were almost five times more likely to report PTSD after the wildfire. There has been a lot of study conducted on the impact of previous mental health issues on PTSD. Beliveau and colleagues conducted a study with 1820 respondents who supported the mission in Afghanistan [67]. The results reveal that a history disorder before deployment was associated with trauma related to deployment. Over the previous year, those with a predeployment history of depression experienced the greatest average marginal effect of deployment-related PTSD [67]. One possibility is that genetic factors play a role in the development of both depression and PTSD. Family history is believed to be a crucial risk factor for depression development. PTSD may also have a hereditary susceptibility according to research [68]. As a result, it stands to reason that genetics may potentially play a role in the appearance of the two conditions. Another explanation is that people with depression are more prone to have traumatic experiences than people without depression, which, in turn, raises the chance of the emergence of PTSD [69].

A lack of or insufficient family support has also been identified as a risk factor for the development of PTSD following the FMM wildfire. Respondents who reported receiving little or no support from family after the wildfire were 11 times more likely to experience PTSD symptoms than those who reported receiving moderate-to-substantial family support. Family supports, which are consistent with our findings, have always been recognized as a protective solid or resource factor in the aftermath of trauma. Support, especially from family members, can reduce both exposure to natural disasters and the negative psychological repercussions of natural disaster exposure [70,71,72,73]. Similar conclusions have been found from other research, such as Chan’s research among 492 survivors of Hurricane Katrina [72]; Thabet’s research on 412 children exposed to war trauma; Dalgleish’s longitudinal study on crisis support in the aftermath of the MS Herald of Free Enterprise tragedy [74]; and Johnson’s research on psychological issues among courthouse shooting victims [75]. Many neurocognitive systems and genetic mechanisms have been linked to family support and human resilience after the disaster [76]. One explanation is about oxytocin. Previous research indicates that oxytocin’s anxiolytic and prosocial effects appear associated with greater prefrontal cortex activity and lower amygdala activity [77,78]. As a result, decreased physiological responsiveness to stress, particularly chronic stress, has been linked to improved psychological and physical well-being [79].

Furthermore, our findings revealed that individuals who were willing to obtain mental health counselling were more than four times more likely to suffer from PTSD than those who were not. It is consistent with findings in the study of PTSD in Fort McMurray residents six months following a wildfire, which revealed a high correlation between “seeking counselling after the wildfire” and “receiving counselling after the wildfire” [30]. This finding suggested that those who obtained counselling may have undergone intense distress and mental health difficulties in the six months following the disaster [30]. According to a Canadian survey, more than 91.5% of people with possible current mental health disorders in the general population have received counselling or therapy [80]. Therefore, it is understandable for respondents of Fort McMurray who would suffer from PTSD-related symptoms to seek counseling after the disaster. Future research with more concrete output on events before and after disasters are required to demonstrate any negative relationship between seeking counseling and mental health consequences. Some authors actively advocate for the development of early mental health intervention programs following disasters, despite the fact that early diagnosis remains a substantial difficulty [15].

Dealing with a disaster is known to be related to a person’s social support, resilience, and coping strategies; thus, it may not be the mere exposure to a disaster but rather the cognitive–emotional appraisal with such an event that leads to short- and long-term psychological consequences [18]. 

Rebuilding devastated communities can be extremely challenging when many residents suffer from mental health difficulties, which is made worse if health services have been disrupted by the disaster [81]. In this regard, it is necessary to investigate the prevalence of MDD and PTSD and the risk factors that are associated with it in traumatized communities to monitor recovery over time and develop more effective strategies to identify those in need of mental health resources.

## 5. Limitations

Research that evaluates and assesses postdisaster conditions often presents some unavoidable limitations. Therefore, the findings of our study need to be understood in light of these limitations. First, the conditions in Fort McMurray following the wildfire did not allow for a more systematic approach to data collecting. As a result, we were forced to employ convenient sampling techniques. Moreover, because of the limited resources available at the time, we relied on volunteer self-reports. As a result, no formal diagnosis could be made, so our analysis relies on a likely MDD and PTSD diagnosis. In addition, because the study was carried out five years after the disaster, the recall bias cannot be overlooked, particularly for the questions related to the wildfire. The mode of administration of the questionnaire has implications on the validity of the study design. Self-administered surveys have low response rates. For instance, very few people would be interested in responding to a mail survey when there is no incentive. This may have implications on the characteristics of the sample in relation to the population. Our assumption is that failure to respond was a random event, thus mitigating potential selection bias. Responder bias is also a possibility given that the researcher has no way of verifying if the information provided is accurate. However, we assume that such occurrences (if any) are minimal and random. Additionally, our sample may not represent the general population of Fort McMurray but rather reflect the residents who were reachable via our recruitment medium (emails sent by intermediaries) at the time of the pandemic. Moreover, it is possible that individuals with higher symptomology would be more likely to respond to our survey. Furthermore, data was collected during the COVID-19 pandemic, which might have also influenced the presence of MDD and PTSD symptoms and respondents. As all the study participants were exposed to the pandemic, we did not include COVID-19 pandemic as a variable in this study. Lastly, with a sample size of 186 rather than the 1058 we had expected for, our estimations of the prevalence rates for MDD and PTSD had a margin of error of 7.18% at 95% confidence intervals, rather than the 3% we had anticipated.

## 6. Conclusions

Our study evaluated the prevalence rates of MDD and PTSD using self-administered scales among residents of Fort McMurray five years after the disaster and determined demographic, clinical, and other risk factors of likely MDD and PTSD in the respondents. Our findings suggested that 5 years following the Fort McMurray wildfire and the subsequent evacuation in 2016, 45.0% of participants still experience likely MDD, and 39.6% of the respondents suffered from probable PTSD. Study results indicate that four variables, unemployment, previous diagnosis of MDD, history of hypnotic medication, and willingness to receive mental health counseling significantly predicted moderate to severe depression amongst respondents. Three independent variables, previous diagnosis of depression, willingness to receive mental health counseling, and having only limited or no support from family contributed to the model for predicting likely PTSD among respondents. Consistent with previous studies, perceived family support was one of the solid protective factors for developing possible PTSD after a traumatic event such as a wildfire. These findings are consistent with the published literature. More extensive research is needed in the future to examine the possible relationship between counselling and likely MDD and PTSD symptoms. Moreover, policy makers need to develop preparedness plans for disasters in the future which provide broad mental health resilience education to the residents in high-risk areas. Continued assessment of the residents and provision of cost-conservative, self-directed, and location-independent interventions such as daily supportive text messaging [9,10,36,46,73,82,83,84,85,86,87] as well as other interventions available during crisis time, such as psychological first aid (PFA) [18], will enhance the available literature and help guide policy makers to develop effective strategies to assist populations with similar problems in the future.

## Figures and Tables

**Table 1:** Demographic information, clinical characteristics, and support received by respondents.

Variables	In a Relationship n (%)	Not in a Relationship n (%)	Total n (%)
**Gender**			
Male	17 (12.9)	10 (18.5)	27 (14.5)
Female	115 (87.1)	44 (81.5)	159 (85.5)
**Age categories**			
≤40 y	62 (47.0)	26 (48.1)	88 (47.3)
>40 y	70 (53.0)	28 (51.9)	98 (52.7)
**Employment status**			
Employed	125 (94.7)	50 (92.6)	175 (94.1)
Unemployed	7 (5.3)	4 (7.4)	11 (5.9)
**Employment place**			
School boards	65 (52.4)	22 (44.0)	87 (50.0)
Healthcare industry	9 (7.3)	1 (2.0)	10 (5.7)
Keyano College	14 (11.3)	6 (12.0)	20 (11.5)
Oil Sands industry	8 (6.5)	5 (10.0)	13 (7.5)
Municipal or government agency	11 (8.9)	2 (4.0)	13 (7.5)
Other	17 (13.7)	14 (28.0)	31 (17.8)
**History of mental health diagnosis from a health professional prior to 2016 wildfire**			
Depressive disorder	37 (28.0)	21 (38.9)	58 (31.2)
Bipolar disorder	3 (2.3)	3 (5.6)	6 (3.2)
Anxiety disorder	54 (40.9)	24 (44.4)	78 (41.9)
Schizophrenia	0 (0.0)	0 (0.0)	0 (0.0)
Personal disorder	0 (0.0)	2 (3.7)	2 (1.1)
Others	12 (9.1)	5 (9.3)	17 (9.1)
No mental health diagnosis	69 (52.3)	21 (38.9)	90 (48.4)
**History of psychotropic medications prior to 2016 wildfire**			
Antidepressants	34 (25.8)	25 (46.3)	59 (31.7)
Antipsychotics	2 (1.5)	2 (3.7)	4 (2.2)
Benzodiazepines	3 (2.3)	1 (1.9)	4 (2.2)
Mood stabilizers	5 (3.8)	7 (13.0)	12 (6.5)
Sleeping tablets	12 (9.1)	9 (16.7)	21 (11.3)
Other	2 (1.5)	1 (1.9)	3 (1.6)
Not on psychotropic medication	92 (69.7)	28 (51.9)	120 (64.5)
**Respondents who received MH counseling in the past year**	48 (36.4)	24 (44.4)	72 (38.7)
**Respondents who would like to receive MH counseling**	66 (50.0)	32 (59.3)	98 (52.7)
**Respondents who resided in Fort McMurray during the 2016 wildfire**	124 (93.9)	52 (96.3)	176 (94.6)
**Area of residence during wildfire**			
Not wildfire areas	104 (83.9)	41 (78.8)	145 (82.4)
Wildfire areas	20 (16.1)	11 (21.2)	31 (17.6)
**Housing status prior 2016 wildfire**			
Own home	106 (80.3)	30 (55.6)	136 (73.1)
Renting	26 (19.7)	24 (44.4)	50 (26.9)
**Housing status now**			
Own home	113 (85.6)	32 (59.3)	145 (78.0)
Renting	19 (14.4)	22 (40.7)	41(22.0)
**Where did you live on the 3rd of May when there was an order to evacuate?**			
In Fort McMurray	116 (913)	43 (86.0)	159 (89.8)
Other	11 (8.7)	7 (14.0)	18 (10.2)
**Respondents reported witnessing the burning of homes or structures by the wildfire**	112 (88.2)	36 (72.0)	148 (83.6)
**Fearful for your life/family/friends during evacuation?**	115 (90.6)	43 (86.0)	158 (89.3)
**Frequency of watching TV on the wildfire devastation**			
Daily	105 (82.7)	37 (74.0)	142 (80.2)
Less than daily	15 (11.8)	8 (16.0)	23 (13.0)
Respondents did not watch the TV images of the devastation	7 (5.5)	5 (10.0)	12 (6.8)
**Frequency of reading newspapers/articles on the wildfires**			
Daily	110 (86.6)	42 (84.0)	152 (85.9)
Less than daily	13 (10.2)	6 (12.0)	19 (10.7)
Respondents did not read newspapers or articles about the devastation	4 (3.1)	2 (4.0)	6 (3.4)
**Did you lose property because of the wildfire in Fort McMurray?**			
Home was destroyed by the wildfire	19 (14.4)	9 (16.7)	28 (15.1)
Home suffered substantial smoke damage	18 (13.6)	4 (7.4)	22 (11.8)
Home suffered slight smoke damage	43 (32.6)	11 (20.4)	54 (29.0)
Car was destroyed by the wildfire	6 (4.5)	1 (1.9)	7 (3.8)
Suffered no loss of property in the wildfire	52 (39.4)	30 (55.6)	82 (44.1)
**Do you live in the same house you lived in before the evacuation order came into effect?**			
Yes	83 (65.9)	21 (42.0)	104 (59.1)
No, I live in a different house even though the previous home was not destroyed by the wildfire	26 (20.6)	20 (40.0)	46 (26.1)
No, I live in a different house because my previous home was destroyed by the wildfire	17 (13.5)	9 (18.0)	26 (14.8)
**Support received from family in relation to the 2016 wildfire**			
Some-to-high level of support	111 (88.1)	40 (81.6)	151 (86.3)
Limited or no support	15 (11.9)	9 (18.4)	24 (13.7)
**Support received from the Government of Alberta in relation to the 2016 wildfire**			
Some-to-high level of support	82 (65.1)	27 (56.3)	109 (62.6)
Limited or no support	44 (34.9)	21 (43.8)	65 (37.4)
**Support received from Red Cross in relation to the 2016 wildfire**			
Some-to-high level of support	94 (74.6)	36(73.5)	130 (74.3)
Limited or no support	32 (25.4)	13 (26.5)	45 (25.7)
**Support received from Insurer in relation to the 2016 wildfire**			
Some-to-high level of support	95 (75.4)	31 (64.6)	126 (72.4)
Limited or no support	31 (24.6)	17 (35.4)	48 (27.6)
**Receiving counseling upon returning to FMM after the 2016 wildfire**			
Yes	23 (18.3)	12 (25.0)	35 (20.1)
No	103 (81.7)	36 (75.0)	139 (79.9)
**Current MDD**			
At most mild depression	70 (56.9)	23 (50.0)	93 (55.0)
Moderate to severe depression	53 (43.1)	23 (50.0)	76 (45.0)
**Current PTSD**			
PTSD unlikely	76 (63.3)	23 (52.3)	99 (60.4)
PTSD likely	44 (36.7)	21 (47.7)	65 (39.6)

MH: Mental Health.

**Table 2:** Chi-squared/Fisher exact test of association between the demographic, clinical, and wildfire-related variables and MDD and PTSD.

Variables	Likely PTSD	Chi-Square/ Fisher Exact	*p*-Value	Moderate-to-Severe Depression	Chi-Square/ Fisher Exact	*p*-Value
**Demographic Characteristics**						
Gender		0.65	0.49		0.37	0.65
Male	7 (31.8%)			9 (39.1%)		
Female	58 (40.8%)			67 (45.9%)		
**Age categories**		0.17	0.75		0.82	0.44 *
≤40 y	31 (41.3%)			38 (48.7%)		
>40 y	34 (38.2%)			38 (41.8%)		
**Employment status**		12.81	<0.001 *		8.71	**0.004**
Employed	57 (36.5%)			67 (42.1%)		
Unemployed	8 (100%)			9 (90.0%)		
**Employment place**		7.14	0.21		4.30	0.52
School boards	21 (28.0%)			27 (35.5%)		
Healthcare industry	3 (37.5%)			5 (55.6%)		
Keyano College	8 (40.0%)			10 (50.0%)		
Oil Sands industry	7 (58.3%)			6 (50.0%)		
Municipal/government agency	4 (33.3%)			4 (33.3%)		
Other	14 (50.0%)			15 (51.7%)		
**Relationship**		1.65	0.21		0.65	0.49
In a relationship	44 (36.7%)			53 (43.1%)		
Not in a relationship	21 (47.7%)			23 (50.0%)		
**Clinical Characteristics**						
**History of depressive disorder diagnosed by mental health professional prior to 2016 wildfire**		24.37	**<0.001**		20.69	<0.001 *
No	30 (26.8%)			38 (33.0%)		
Yes	35 (67.3%)			38 (70.4%)		
**History of bipolar disorder diagnosed by mental health professional prior to 2016 wildfire**		0.00	0.99 *		0.06	0.99
No	2 (40.0%)			73 (44.8%)		
Yes	63 (39.6%)			3 (50%)		
**History of anxiety disorder diagnosed by mental health professional prior to 2016 wildfire**		19.49	**<0.001**		14.30	<0.001 *
No	24 (25.3%)			32 (32.7%)		
Yes	41 (59.45)			44 (62.0%)		
**History of personality disorder diagnosed by mental health professional prior to 2016 wildfire**		66	0.99		0.82	0.99 *
No	65 (39.9%)			76 (45.2%)		
Yes	0 (0.0%)			0 (0.00%)		
**History of other disorder diagnosed by mental health professional prior to 2016 wildfire**		0.00	0.99		0.18	0.79
No	59 (39.6%)			68 (44.4%)		
Yes	6 (40.0%)			8 (50.0%)		
**Never received a mental health diagnosis from a health professional prior to 2016 wildfire**		16.47	**<0.001**		13.50	<0.001 *
No	19 (23.8%)			25 (30.5%)		
Yes, received MH Dx	46 (54.8%)			51 (58.6%)		
**History of Antidepressant medications prior to 2016 wildfire**		9.18	**<0.01**		6.55	**0.013**
No	36 (31.9%)			44 (38.3%)		
Yes	29 (56.9%)			32 (59.3%)		
History of Antipsychotic Medications **prior** to 2016 wildfire		0.09	0.99 *		0.04	0.99 *
No	64 (39.5%)			74 (44.8%)		
Yes	1 (50.0%)			2 (50.0%)		
History of **Benzodiazepine** Medications prior to 2016 wildfire		6.25	0.02 *		5.01	0.039 *
No	61 (38.1%)			72 (43.6%)		
Yes	4 (100.0%)			4 (100.0%)		
**History of Mood Stabilizer Medications prior to 2016 wildfire**		1.01	0.49		0.436	0.55
No	60 (38.7%)			70 (44.3%)		
Yes	5 (55.6%)			6 (54.5%)		
**History of Sleeping Tablets prior to 2016 wildfire**		10.76	**<0.001**		13.32	<0.001 *
No	52 (35.4%)			60 (40.0%)		
Yes	13 (76.5%)			16 (84.2%)		
**Not on any medication for mental health concerns prior to 2016 wildfire**		7.16	**0.01**		7.61	0.007 *
No	34 (32.1%)			40 (37.0%)		
Yes, on MH Mx	31 (53.4%)			36 (59.0%)		
**Respondents received MH counseling in the past year**		20.85	**<0.001**		6.87	0.011 *
No	27 (26.2%)			38 (37.1%)		
Yes	38 (62.3%)			37 (57.8%)		
**Respondents would like to receive MH counseling**		29.24	**<0.001**		24.48	<0.001 *
No	14 (17.9%)			20 (25.0%)		
Yes	51 (59.3%)			56 (62.9%)		
**Fort McMurray Wildfire-Related Characteristics**						
**Where did you live on the 3rd May evacuation for the 2016 wildfires?**		1.30	0.28		0.02	0.99
In Fort MacMurray	57 (38.3%)			69 (44.8%)		
Other	8 (53.3%)			7 (46.7%)		
**Did you reside in Fort McMurray during the 2016 wildfire?**		0.58	0.54		0.06	0.99
No	6 (50.0%)			5 (41.75)		
Yes	59 (38.8%)			71 (45.2%)		
**Area of residence in wildfire**		3.66	0.16		3.38	0.19
0–1.0 properties destroyed per km^2^	23 (34.8%)			29 (42.0%)		
1.1–50.0 properties destroyed per km^2^	15 (33.3%)			18 (39.1%)		
50.1–300.0 properties destroyed per km^2^	21 (51.2%)			24 (57.1%)		
**Type of residence prior to wildfire**		0.13	0.85		0.04	0.86
Own home	49 (38.9%)			57 (44.5%)		
Renting	16 (42.1%)			19 (46.3%)		
**Type of residence now**		0.04	0.99		0.25	0.70
Own home	52 (40.0%)			62 (45.9%)		
Renting	13 (38.2%)			14 (41.2%)		
**Did you witness the burning of any homes or structures in Fort McMurray?**		0.72	0.51		0.09	0.83
No	8 (32.0%)			11 (42.3%)		
Yes	57 (41.0%)			65 (45.5%)		
**Fearful for your life or the lives of your friends or family in evacuation**		7.61	**0.01**		4.95	0.029 *
No	2 (10.5%)			4 (21.1%)		
Yes	63 (43.4%)			72 (48.0%)		
**Frequency of watching TV on the wildfire devastation**		3.62	0.16		0.41	0.84
Daily	55 (42.3%)			61 (45.2%)		
<Daily	5 (21.7%)			11 (47.8%)		
I did not watch the TV images of the devastation	5 (45.5%)			4 (36.4%)		
**Frequency of reading newspapers/articles on the wildfires**		4.21	0.17 *		2.03	0.43 *
Daily	60 (42.9%)			67 (46.2%)		
<Daily	4 (22.2%)			8 (44.4%)		
I did not read newspaper and internet articles related to the devastation	1 (16.7%)			1 (16.7%)		
**Lost property (home completely destroyed)**		1.39	0.28		1.46	0.29
No	52 (37.7%)			61 (43.0%)		
Yes	13 (50.0%)			15 (55.6%)		
**Lost property (home suffered substantial smoke damage)**		3.09	**0.10**		0.00	0.99
No	53 (37.1%)			66 (44.9%)		
Yes	12 (57.1%)			10 (45.5%)		
**Lost property (home suffered slight smoke damage)**		0.38	0.61		0.15	0.74
No	43 (38.1%)			51 (44.0%)		
Yes	22 (43.1%)			25 (47.2%)		
**Lost property (car was completely destroyed)**		0.94	0.44 *		0.44	0.70 *
No	61 (38.9%)			72 (44.4%)		
Yes	4 (57.1%)			4 (57.1%)		
**Suffered no loss of properties**		7.05	**0.01**		1.71	0.21
No, did not lose	18 (27.3%)			26 (38.8%)		
Yes, lose	47 (48.0%)			50 (49.0%)		
**Do you live in the same house you lived in before the evacuation order came into effect?**		6.72	**0.03**		4.49	0.11
Yes	31 (31.6%)			39 (39.4%)		
No, I live in a different house even though my previous home was not destroyed by the fire.	23 (53.5%)			22 (48.9%)		
No, I live in a different house because my previous home was destroyed by the fire.	11 (47.8%)			15 (62.5%)		
**Support received from Family in relation to the 2016 wildfire**		14.62	**<0.001**		7.25	**0.008**
Some-to-high level of support	48 (34.3%)			59 (41.3%)		
Limited or no support	17 (77.3%)			17 (70.8%)		
**Support received from the Government of Alberta Insurer in relation to the 2016 wildfire**		2.67	0.14		6.27	**0.02**
Some-to-high level of support	36 (35.3%)			40 (38.1%)		
Limited or no support	29 (48.3%)			36 (58.1%)		
**Support received from Red Cross in relation to the 2016 wildfire**		4.03	**0.06**		4.44	**0.05**
Some-to-high level of support	44 (35.8%)			51 (40.8%)		
Limited or no support	21 (53.8%)			25 (59.5%)		
**Support received from Insurer in relation to the 2016 wildfire**		3.71	**0.07**		2.00	0.17
Some-to-high level of support	42 (35.6%)			61 (42.1%)		
Limited or no support	23 (52.3%)			15 (54.3%)		
**Did you receive any counseling when you returned to Fort McMurray after the wildfires?**		2.11	0.16		0.000	0.99
**Yes**	16 (51.6%)			15 (45.5%)		
**No**	49 (37.4%)			61 (45.5%)		

* Fisher Exact test.

**Table 3:** Logistic regression predicting the likelihood of residents presenting with MDD.

Predictor	B	S.E	Wald	df	*p*-Value	Odds Ratio	95% CI for Odds Ratio	
							Upper Lower	
**Unemployed**	**2.52**	**1.19**	**4.51**	**1**	**0.03**	**12.39**	**1.21**	**126.37**
**History of depressive disorder diagnoses by health care professional prior to the 2016 wildfire**	1.50	0.54	7.80	1	**0.01**	4.50	1.57	12.92
**History of anxiety disorder diagnoses by health care professional** **prior to the 2016 wildfire**	0.51	0.52	0.99	1	0.32	1.67	0.61	4.60
**Received mental health counseling in the past year**	−0.70	0.50	1.95	1	0.16	0.50	0.19	1.32
**Would like to receive mental health counseling**	1.59	0.47	11.43	1	**<0.01**	4.90	1.95	12.31
**On sleeping tablets**	1.66	0.84	3.90	1	**0.048**	5.27	1.01	27.39
**Not on any medication for a mental health concern**	−0.71	0.59	1.50	1	0.22	0.49	0.16	1.54
**Feel fearful for my life or the lives of my friends and family during evacuation**	0.44	0.73	0.37	1	0.54	1.56	0.37	6.49
**Received limited or no support from family in relation to the 2016 wildfire**	0.83	0.66	1.59	1	0.20	2.30	0.63	8.38
**Received limited or no support from the Red Cross in relation to the 2016 wildfire**	−0.09	0.58	0.03	1	0.88	0.91	0.29	2.86
**Received limited or no support from Government of Alberta in relation to the 2016 wildfire**	0.50	0.48	1.11	1	0.29	1.65	0.65	4.20
**Constant**	−2.15	0.72	9.00	1	0.00	0.12		

CI: confidence interval. S.E.: standard error. df: degree of freedom.

**Table 4:** Logistic regression predicting the likelihood of residents presenting with PTSD.

Predictor	B	S.E	Wald	df	*p*-Value	Odds Ratio	95% CI for Odds Ratio	
							Upper Lower	
**History of depressive disorder diagnoses by health care professional prior to the 2016 wildfire**	1.50	0.60	6.35	1	**0.01**	4.49	1.40	14.44
**History of anxiety disorder diagnoses by health care professional prior to the 2016 wildfire**	0.74	0.58	1.62	1	0.20	2.10	0.67	6.56
**Received mental health counseling in the past year prior to the 2016 wildfire**	0.46	0.51	0.79	1	0.37	1.58	0.58	4.32
**Would like to receive mental health counseling**	1.47	0.53	7.67	1	**0.01**	4.35	1.54	12.34
**On sleeping tablets prior to the 2016 wildfire**	1.58	0.96	2.73	1	0.10	4.86	0.75	31.70
**Not on any medication for a mental health concern prior to the 2016 wildfire**	−1.26	0.70	3.30	1	0.07	0.28	0.07	1.10
**Do you live in the same house as before the evacuation?**								
Yes			2.73	2	0.26			
No, even though previous home was not destroyed by the fire	0.80	0.54	2.24	1	0.13	2.24	0.78	6.40
No, because previous home was destroyed by the fire	−0.30	0.67	0.19	1	0.66	0.74	0.20	2.77
**Feel fearful for my life or the lives of my friends and family during evacuation**	1.53	1.14	1.79	1	0.18	4.615	0.49	43.32
**Home suffered substantial smoke damage because of the wildfires**	0.93	0.72	1.70	1	0.19	2.54	0.63	10.34
**Suffered no loss of property in the fire**	0.93	0.50	3.41	1	0.07	2.54	0.95	6.84
**Received limited or no support from family in relation to the 2016 wildfire**	2.40	0.89	7.24	1	**0.01**	11.01	1.92	63.20
**Received limited or no support from the Red Cross in relation to the 2016 wildfire**	−0.007	0.64	0.00	1	0.99	0.99	0.28	3.50
**Received limited or no support from insurer in relation to the 2016 wildfire**	0.09	0.57	0.03	1	0.87	1.09	0.36	3.31
**Constant**	−4.56	1.21	14.17	1	0.00	0.01		

CI: confidence interval. S.E.: standard error. df: degree of freedom.

## Data Availability

Data are available upon reasonable request to the submitting author.
